# Combined functional strength training and progressive muscle relaxation in informal caregivers of people with dementia: protocol for a randomized controlled trial

**DOI:** 10.3389/fpsyg.2026.1944170

**Published:** 2026-09-17

**Authors:** Alba Sánchez-Gil, Juan Luis Sánchez-González, Marta Gómez-Mateos, Vanesa Santos-Rodríguez, Raquel García-Santelesforo, Jesús Pérez, Fátima Pérez-Robledo, Enrique Pérez-Sáez, Andrea Calleja-Caballero

**Affiliations:** 1 Centro de Referencia Estatal de atención a personas con enfermedad de Alzheimer y otras demencias, Imserso, Salamanca, Spain; 2 Departamento de Medicina, Universidad de Salamanca, Instituto de Investigación Biomédica de Salamanca (IBSAL), Salamanca, Spain; 3 Departamento de Enfermería y Fisioterapia, Universidad de Salamanca, Instituto de Investigación Biomédica de Salamanca (IBSAL), Salamanca, Spain

**Keywords:** anxiety, caregivers, dementia, muscle relaxation, physical functional performance, resistance training

## Abstract

**Background:**

Informal caregivers of people living with dementia are exposed to sustained physical and psychological demands that may increase their risk of functional decline, anxiety, and caregiver burden. While exercise and relaxation interventions have shown independent benefits, evidence on structured multicomponent programs integrating both physical and psychological approaches remains limited. This study protocol describes a randomized controlled trial designed to evaluate the effectiveness of a combined functional strength training and progressive muscle relaxation program in informal caregivers.

**Methods:**

A prospective, parallel-group randomized controlled trial will be conducted in informal caregivers of people with dementia. Sixty-two informal caregivers will be randomly allocated (1:1) to either a combined intervention group (functional strength training plus progressive muscle relaxation) or a comparison group receiving progressive muscle relaxation alone. The intervention will last 10 weeks. The combined intervention group will attend two weekly sessions (one strength training and one relaxation session), whereas the comparison group will attend one weekly relaxation session. Lower-extremity physical performance, assessed using the total Short Physical Performance Battery (SPPB) score at 10 weeks, will be the primary outcome. Secondary outcomes will include handgrip strength, anxiety, caregiver burden, adherence, and safety. Outcomes will also be assessed at the 3-month follow-up. Analyses will follow the intention-to-treat principle.

**Discussion:**

This trial will provide evidence on the effectiveness, feasibility, and safety of a structured multicomponent intervention targeting both physical function and psychological burden in informal caregivers. The findings may contribute to the development of comprehensive and replicable caregiver support programs in real-world health and social care settings.

**Clinical trial registration:**

ClinicalTrials.gov, Identifier: NCT07388186.

## 1 Introduction

Alzheimer’s disease and related dementias are major contributors to disability and dependency among older adults, placing a substantial burden on informal caregivers, who provide most of the long-term care. This sustained caregiving role is associated with adverse physical and psychological outcomes, including increased anxiety, depressive symptoms, and caregiver burden (Fink et al., 2025). Prolonged exposure to caregiving-related stress may also lead to physiological dysregulation, contributing to declines in overall health and well-being (Toleuov et al., 2025; Brites et al., 2025).

Frailty, defined as a state of increased vulnerability due to reduced physiological reserves, has emerged as a relevant but underexplored condition among informal caregivers (Dent et al., 2019). Evidence suggests that caregiving may be associated with greater frailty, with caregivers of people with dementia showing higher frailty index scores than matched non-caregiver controls (Canevelli et al., 2022). In a large European study of co-residential caregivers aged 50 years and older, the prevalence of frailty reached 29.3% among female caregivers in Southern Europe, which included Spain (Barbosa et al., 2020). The combination of physical demands and chronic emotional strain may contribute to physiological dysregulation and functional decline (Rohleder, 2019), potentially increasing the risk of disability and compromising both caregiver health and the quality of care provided. Although interventions such as relaxation techniques and physical exercise have shown benefits in reducing psychological distress and improving physical capacity, most approaches target these domains separately, and evidence on integrated, multicomponent interventions remains limited (He et al., 2022; Turan et al., 2025; Cadore and Izquierdo, 2015). In this context, functional strength training may help address the physical demands associated with caregiving by targeting functional capacity and muscle strength, while progressive muscle relaxation may address psychological demands associated with anxiety, stress, and caregiver burden. The combination of both approaches may therefore provide a more comprehensive strategy to address the interconnected physical and psychological dimensions of caregiver health.

There is a need for feasible and replicable interventions that address both physical and psychological components within real-world care settings (Damschroder et al., 2009). The development of structured, low-cost, and easily replicable programs is essential to facilitate implementation within community resources and specialized care centers, as well as their integration into caregiver support strategies (Sörensen et al., 2006). The present article describes the study protocol, which aims to evaluate the effects of the intervention on caregivers’ physical performance and psychological health outcomes while providing a methodologically robust and replicable intervention model developed within a real-world health and social care setting.

## 2 Objectives and hypotheses

### 2.1 Primary objective

To evaluate the effect of adding functional strength training to progressive muscle relaxation, compared with progressive muscle relaxation alone, on lower-extremity physical performance measured using the Short Physical Performance Battery at 10 weeks.

### 2.2 Secondary objectives

1. To evaluate the effect of the intervention on handgrip strength.
2. To evaluate the effect of the intervention on anxiety symptoms.
3. To evaluate the effect of the intervention on caregiver burden.
4. To evaluate whether the intervention effects are maintained at the 3-month follow-up.
5. To describe intervention adherence, safety, tolerability, and retention.

### 2.3 Hypothesis

Informal caregivers allocated to the combined intervention are expected to demonstrate a greater improvement in total SPPB score at 10 weeks than those receiving progressive muscle relaxation alone.

## 3 Methods

### 3.1 Study design

A prospective, parallel-group randomized controlled trial will be conducted to evaluate the effects of a combined functional strength training and progressive muscle relaxation program compared with progressive muscle relaxation alone in informal caregivers of people with dementia. An overview of the study design, intervention groups, assessment time points, and main outcomes is presented in Figure 1.

**Figure 1:**
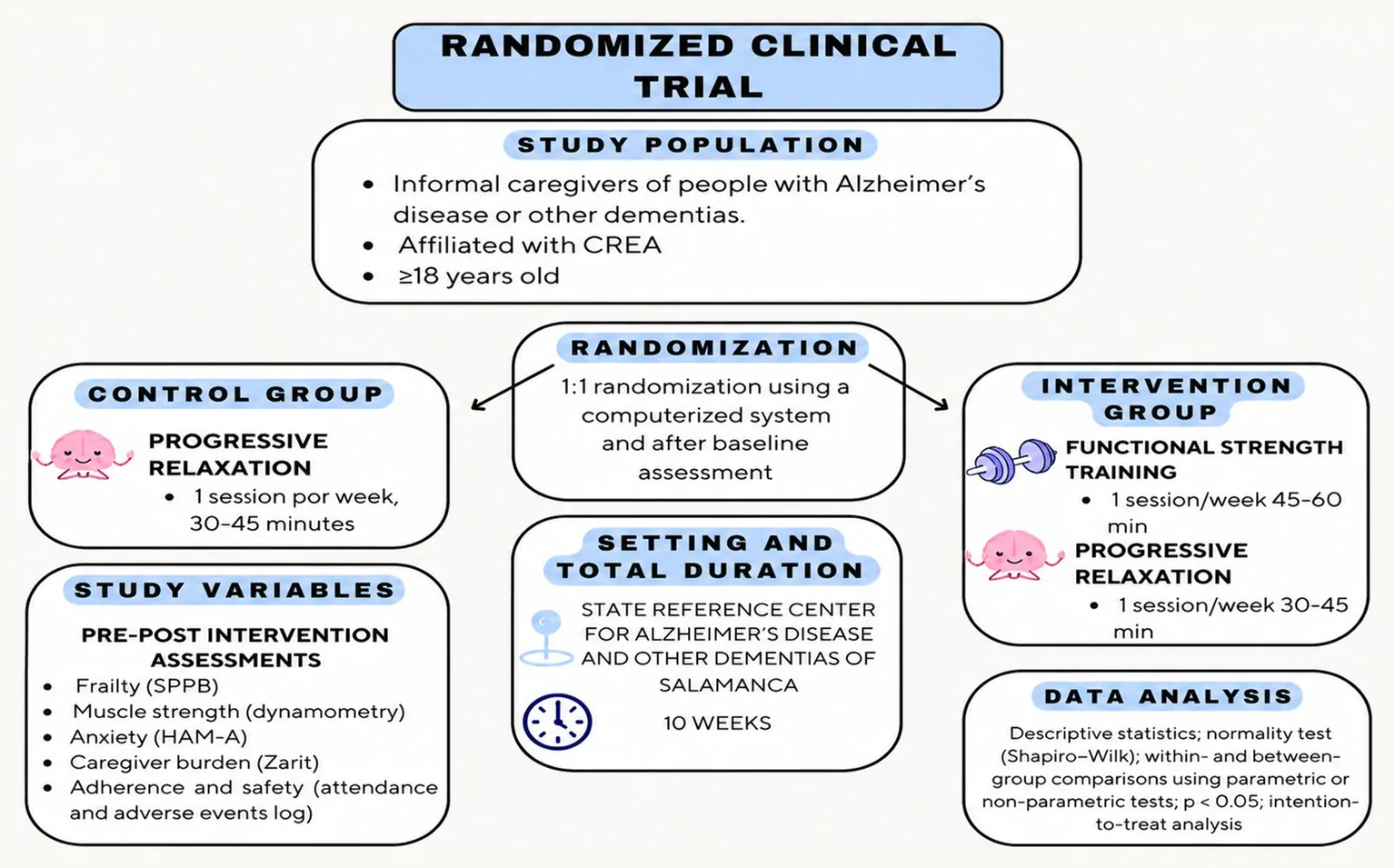
Overview of the study design, intervention groups, assessment time points, and main outcomes.

The study will adhere to the Consolidated Standards of Reporting Trials (Moher et al., 2012) guidelines for randomized clinical trials, and the intervention protocol is reported in accordance with the Standard Protocol Items: Recommendations for Interventional Trials (Chan et al., 2013) statement. The study is scheduled to be conducted between March 2026 and December 2026. The intervention is described in accordance with the Template for Intervention Description and Replication (TIDieR) checklist (see Supplementary Materials S1, S2) to ensure transparency, reproducibility, and completeness of reporting.

All procedures performed in this study will comply with the ethical standards of the relevant national and institutional committees on human experimentation and with the Declaration of Helsinki of 1975, revised in 2008. The study protocol has been reviewed and approved by the Research Ethics Committee of the University of Salamanca (approval number: 1468). The trial is registered at ClinicalTrials.gov (ID: NCT07388186).

All participants will receive detailed information about the study objectives and procedures and will provide written informed consent prior to enrolment. Participants’ privacy will be respected in accordance with current data protection regulations, and anonymity will be ensured when study data are reported in scientific publications. Compliance with applicable data protection and confidentiality regulations will be ensured through the assignment of numerical identification codes to each participant in order to preserve anonymity.

The risk of adverse events related to the proposed interventions is considered minimal. Nevertheless, any adverse event occurring during or after the study period will be closely monitored and documented by the research team as part of routine intervention follow-up. Serious adverse events will be systematically documented and promptly reported to the principal investigators within 24 h of detection. When necessary, appropriate measures will be implemented to ensure participant safety and well-being, including temporary modification of the intervention or withdrawal of the participant from the study.

### 3.2 Setting

The study will be conducted at the Centro de Referencia Estatal de Atención a Personas con Enfermedad de Alzheimer y otras Demencias (CREA) in Salamanca, Spain, a national reference center under the Instituto de Mayores y Servicios Sociales (Imserso), specializing in care provision, research, and innovation in the field of Alzheimer’s disease and related dementias. In addition, the center develops activities and programs aimed at supporting informal caregivers within a comprehensive care model that addresses both people living with dementia and their caregiving environment.

Within this study, CREA will serve as the recruitment and intervention setting, facilitating access to informal caregivers linked to the center and providing appropriate facilities for the planned assessments and intervention sessions. Intervention delivery and outcome assessments will be conducted by the research team.

Conducting the study in this setting will allow the evaluation not only of the intervention’s effectiveness but also of its feasibility, safety, and real-world applicability, thereby supporting the translation of study findings into routine clinical and community-based practice.

### 3.3 Participants

The study population will consist of informal caregivers of people with dementia linked to the CREA center in Salamanca. An informal caregiver will be defined as an individual who provides regular, unpaid care and support to a relative or close person diagnosed with Alzheimer’s disease or another form of dementia.

Baseline caregiver characteristics will include age, sex, educational level, employment status, relationship to the person with dementia, co-residence, duration of caregiving, weekly caregiving hours, and availability of informal or formal support, relevant chronic conditions, medication use, and usual physical activity.

Characteristics of the person receiving care will include the presence and type of dementia diagnosis (e.g., Alzheimer’s disease, vascular dementia, Lewy body dementia, frontotemporal dementia, mixed or other dementia), the date or year of diagnosis when available, functional dependency, relevant behavioral and psychological symptoms. Behavioral and psychological symptoms will be recorded based on information provided by the informal caregiver and, when available, information from the person’s clinical records. Additional characteristics will include use of formal care services and major changes in care arrangements during the study.

Major changes occurring during follow-up, including hospitalization, institutionalization, changes in caregiving hours, initiation of formal respite care, or changes in caregiver medication or exercise behaviour, will be recorded.

#### 3.3.1 Inclusion criteria

Participants will be eligible if they meet all the following inclusion criteria:

1. Be aged 18 years or older.
2. Be an informal caregiver of a person diagnosed with Alzheimer’s disease or another form of dementia.
3. Provide at least 10 h of informal care per week.
4. Be able to attend the intervention sessions throughout the study period.
5. Be able to understand and follow the study instructions.
6. Provide written informed consent before participation.

#### 3.3.2 Exclusion criteria

Participants will be excluded if they meet any of the following exclusion criteria:

1. Have an acute illness or medical condition that contraindicates participation in strength exercise.
2. Be currently participating in a structured exercise program that could interfere with the study outcomes.
3. Have a severe or uncontrolled psychiatric disorder.
4. Have physical limitations that prevent completion of the functional assessments.

Eligibility for exercise participation will be confirmed through a standardized pre-exercise health screening conducted before the start of the intervention. The screening will include a review of current health status, relevant medical conditions, medication use, recent acute illness or injury, and any symptoms or physical limitations that could contraindicate or require modification of strength exercise or the planned functional assessments. The research team will also review whether participants meet the predefined exclusion criteria related to medical conditions, psychiatric disorders, current structured exercise, and physical limitations. When the screening identifies a potential health risk or uncertainty regarding exercise safety, medical clearance will be requested before participation.

### 3.4 Randomization and blinding

Participants will be randomly allocated in a 1:1 ratio to either the combined intervention group (combined functional strength training and progressive muscle relaxation) or the comparison group (progressive muscle relaxation alone) using a computer-generated randomization sequence with randomly varying block sizes. The block sizes will not be disclosed to the researchers involved in recruitment or enrollment. The anticipated flow of participants through recruitment, randomization, intervention, follow-up, and analysis is shown in Figure 2.

**Figure 2:**
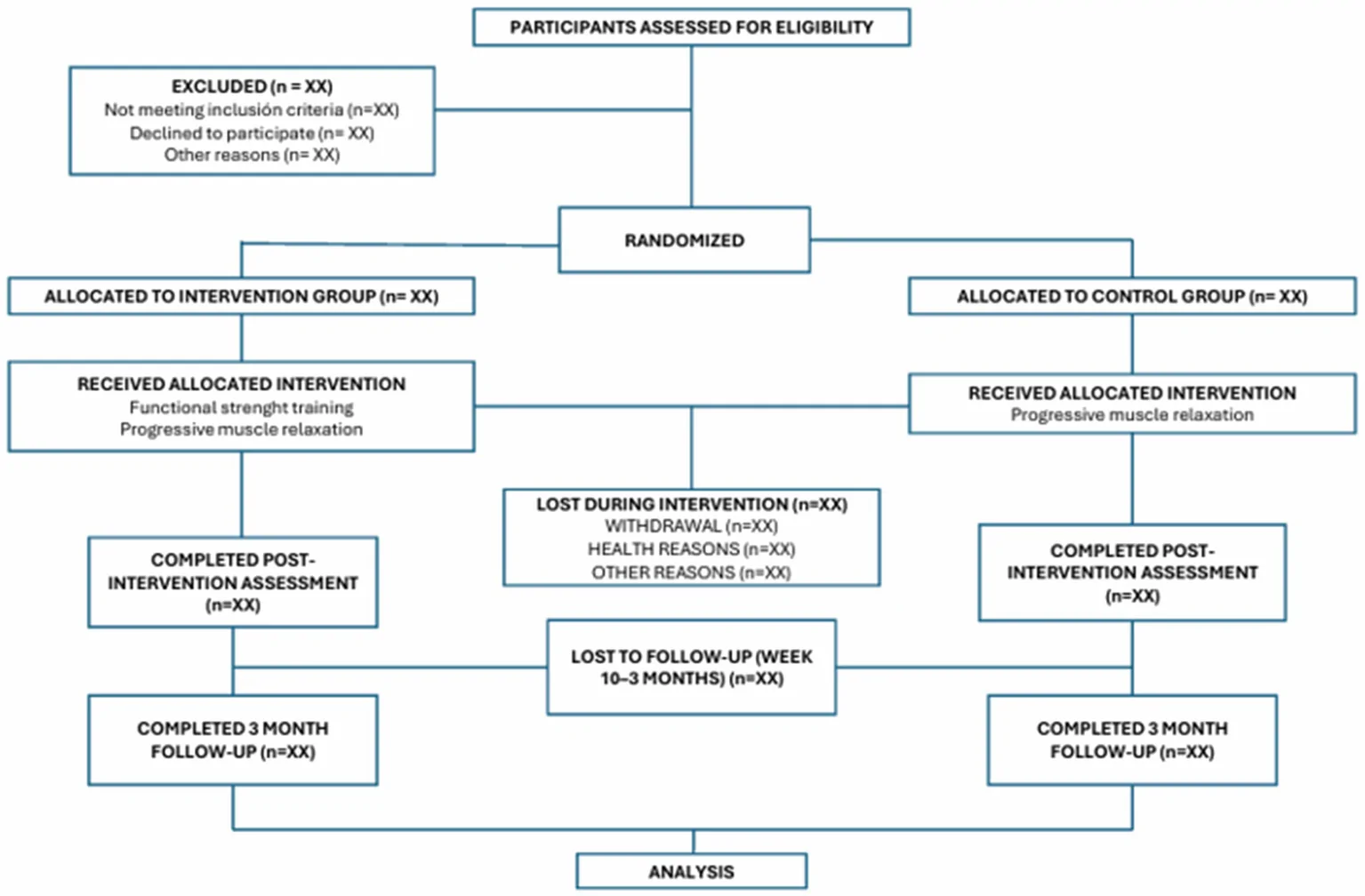
Flow diagram of participant recruitment, randomization, intervention allocation, follow-up, and analysis.

The allocation sequence will be generated by an independent researcher or statistician who will not participate in recruitment, baseline assessment, intervention delivery, or outcome assessment. Group assignments will be placed in sequentially numbered, opaque, sealed, and tamper-evident envelopes. The envelopes will be stored securely and opened in numerical order only after eligibility has been confirmed and all baseline assessments have been completed.

Due to the nature of the intervention, blinding of participants and intervention providers will not be feasible. All outcome assessments will be performed by an independent researcher who will not participate in recruitment, intervention delivery, or any other study procedures that could reveal participants’ group allocation. The assessor will remain blinded to group allocation throughout the assessment period to minimize the risk of measurement bias.

### 3.5 Intervention

Participants enrolled in the study will be allocated to one of the two intervention groups for a 10-week period. The duration of the intervention was selected based on previous caregiver intervention programs, which commonly range from approximately 8 to 12 weeks. A 10-week duration was therefore chosen as an intermediate timeframe, aiming to provide sufficient exposure to the intervention while maintaining feasibility for informal caregivers.

Participants in the combined intervention group will attend two weekly sessions: one session of functional strength training and one session of progressive muscle relaxation. Participants in the comparison group will attend one weekly session of progressive muscle relaxation. All sessions will be conducted at the CREA facilities in Salamanca and will be led and supervised by licensed physiotherapists with at least 2 years of clinical experience in therapeutic exercise and prior training in progressive muscle relaxation techniques.

Both groups will receive structured, face-to-face, group-based interventions designed to ensure participant safety and promote adherence to the program. Sessions will take place in a controlled, accessible, and appropriately adapted environment and will follow a standardized protocol previously defined by the research team.

The functional strength training sessions will focus on improving functional capacity and muscle strength through exercises adapted to each participant’s individual functional level. The progressive muscle relaxation sessions will follow a structured protocol aimed at reducing anxiety, stress, and caregiver burden. The same progressive muscle relaxation protocol will be delivered to both groups, ensuring that both groups receive an active and structured intervention. This design will allow estimation of the additional effect of adding functional strength training to progressive muscle relaxation. However, differences in total therapist contact time and intervention exposure between groups may contribute to the observed between-group effects.

A systematic record of adverse events occurring during the intervention period will be maintained. An adverse event will be defined as any undesired incident or clinical manifestation occurring during the intervention period, regardless of its severity or causal relationship with the program. Before each training session, participants will be asked whether they have experienced any adverse events or new health problems since the previous session. Any reported event will be documented and reported to the research team, and its severity and potential relationship to the intervention will be assessed. When necessary, appropriate measures will be implemented, including temporary modification of the intervention or suspension of the affected participant’s involvement.

Any modifications to the intervention protocol during the study will be documented, including the rationale for the change and its potential impact on intervention delivery. All modifications will be reported in the final study publication to ensure transparency and reproducibility.

#### 3.5.1 Functional strength training

The functional strength training program will be delivered over a 10-week period, with a frequency of one session per week integrated within the combined intervention program. Each session will last approximately 45–60 min and will be conducted face-to-face in a group format under the direct supervision of a physiotherapist experienced in therapeutic exercise.

Sessions will be structured into three phases: warm-up, main phase, and cool-down. The warm-up will last approximately 10 min and will include global joint mobility exercises, progressive muscle activation, and low-intensity tasks aimed at preparing participants for the subsequent workload, with particular emphasis on postural control.

The main phase will focus on functional strength training, prioritizing multi-joint exercises and tasks that replicate activities of daily living, such as sit-to-stand movements, pushing, pulling, step-ups, and balance exercises, including tandem stance. Exercises will primarily target major muscle groups, especially those of the lower limbs and trunk, given their direct relationship with functional capacity and physical performance.

Exercises will be performed using body weight and simple equipment, including elastic resistance bands, light dumbbells, and balls, and will be individually adapted according to each participant’s functional level. Training intensity will be progressively adjusted by modifying the load, number of repetitions, time under tension, or level of support. In general, exercises will be performed in sets of 8–12 repetitions, with 1–3 sets per exercise and appropriate rest periods between sets. Exercise intensity and progression will be guided by participants´ perceived exertion, functional performance, and predefined safety criteria. Progression decisions will be individualized and based on each participant’s tolerance, correct technical execution, and absence of adverse symptoms.

Elastic resistance bands of varying resistance levels, adjustable dumbbells (1–5 kg), stability balls, and standardized seating equipment will be used. All materials will be provided by the research team and maintained in accordance with manufacturer recommendations.

Program progression will be gradual and individualized, with exercise difficulty increased over time according to participant tolerance, safety, and functional improvements. Throughout all sessions, particular attention will be paid to correct technical execution, perceived exertion, and potential signs of fatigue or discomfort, with participant safety prioritized at all times. An overview of the functional strength training program is presented in Figure 3.

**Figure 3:**
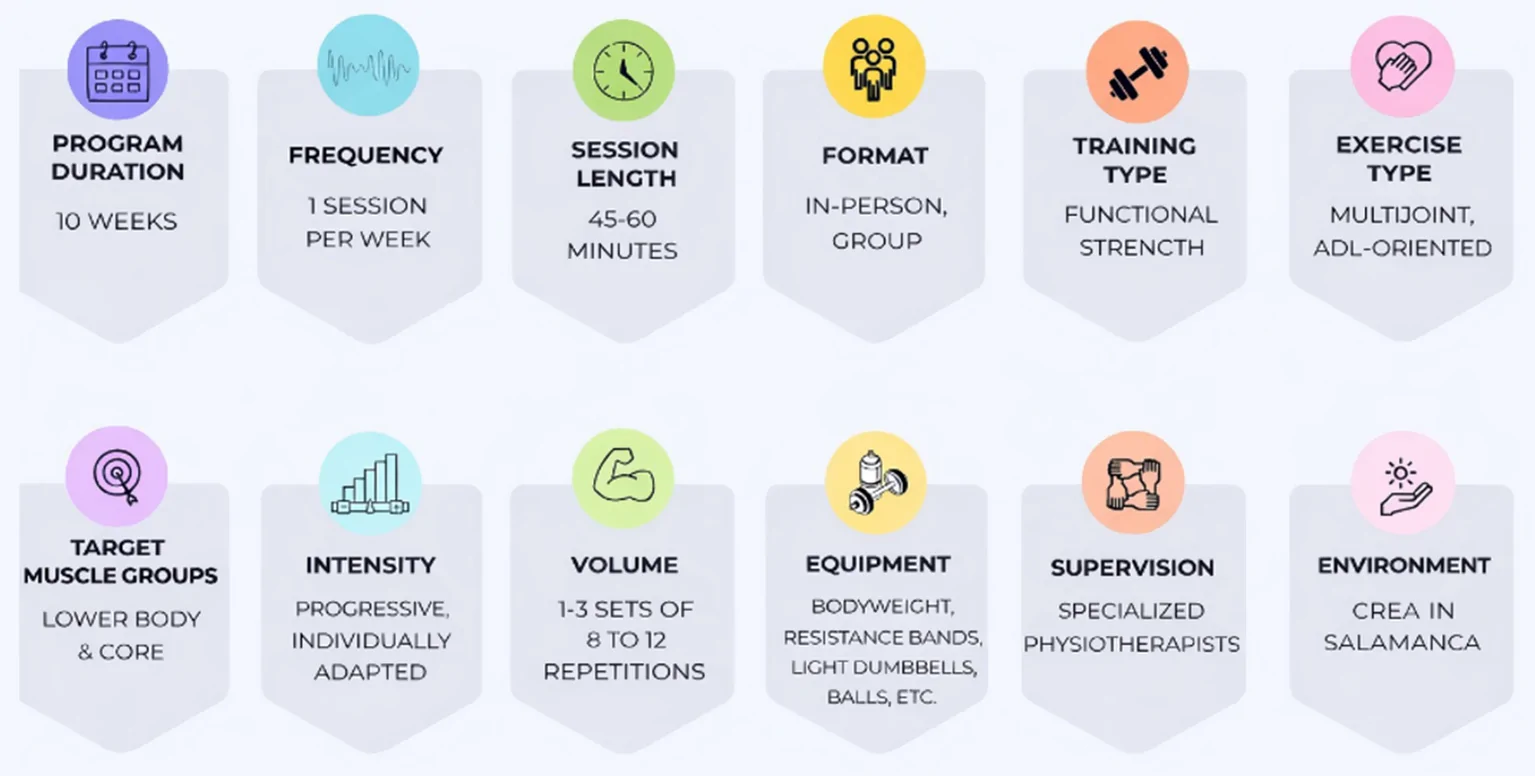
Overview of the 10-week functional strength training program.

#### 3.5.2 Progressive muscle relaxation

The progressive muscle relaxation program will be delivered over a 10-week period, with a frequency of one weekly session in both groups. In the combined intervention group, progressive muscle relaxation will be combined with one additional weekly session of functional strength training, whereas in the comparison group it will be delivered as a standalone intervention. Each session will last approximately 30–45 min and will be conducted face-to-face in a group format under the supervision of a physiotherapist.

The intervention will follow a structured progressive muscle relaxation protocol based on the systematic sequence of contraction and relaxation of the main muscle groups, aiming to enhance body awareness, reduce physiological arousal, and promote stress self-regulation. Sessions will take place in a calm and controlled environment designed to ensure comfort and concentration.

Each session will begin with a brief preparation phase, during which the objectives of the session will be explained, and participants will be guided through slow, controlled breathing followed by the progressive application of the relaxation technique. The sequence will be applied sequentially from distal to proximal muscle groups, respecting appropriate contraction and relaxation times (Bernstein and Borkovec, 1973; Turan et al., 2025).

Throughout all sessions, particular attention will be paid to correct execution of the technique, adapting the pace and instructions to participants’ individual needs. The physiotherapist will provide verbal guidance and monitor participants’ responses to ensure participant safety and intervention tolerability. The program is designed to reduce anxiety, stress, and caregiver-related emotional burden. An overview of the progressive muscle relaxation program is presented in Figure 4.

**Figure 4:**
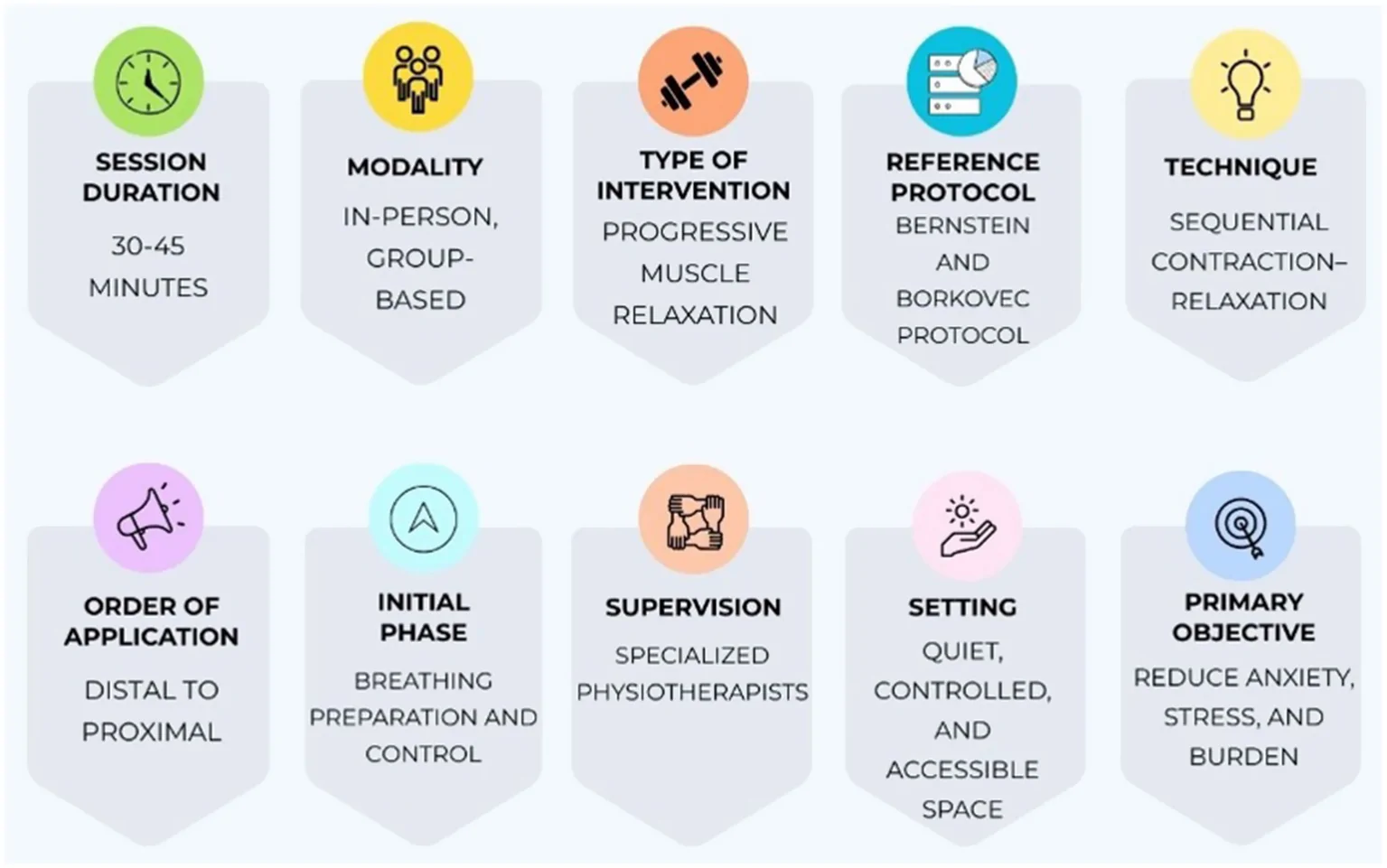
Overview of the 10-week progressive muscle relaxation program.

### 3.6 Outcome measures

Outcome assessments will be conducted at baseline (T0), immediately after completion of the 10-week intervention (T1), and at the 3-month follow-up (T2). The schedule of enrolment, interventions, and assessments is presented in Table 1.

**Table 1:** Schedule of enrolment, interventions, and assessments according to the SPIRIT recommendations.

Activity	Screening	Baseline (T0, week 0)	Intervention (weeks 1–10)	Post-intervention (T1, week 10)	Follow-up (T2, 3 months)
Screening and enrollment					
Eligibility assessment	✓				
Informed consent	✓				
Demographic and clinical characteristics		✓			
Randomization		✓			
Intervention					
Combined Intervention group			Functional strength training (1 session/week) + Progressive muscle relaxation (1 session/week)		
Comparison group			Progressive muscle relaxation (1 session/week)		
Outcome assessments					
Short Physical Performance Battery (SPPB)		✓		✓	✓
Handgrip strength		✓		✓	✓
Hamilton Anxiety Rating Scale (HAM-A)		✓		✓	✓
Zarit Burden Interview (ZBI)		✓		✓	✓
Monitoring					
Attendance/adherence			Continuous		
Adverse events			Continuous	✓	✓

#### 3.6.1 Primary outcome

##### 3.6.1.1 Lower-extremity physical performance

Lower-extremity physical performance will be assessed using the Short Physical Performance Battery (SPPB), a validated instrument comprising three components: standing balance, usual gait speed, and repeated chair stands. Each component is scored from 0 to 4 points, resulting in a total score ranging from 0 to 12, with higher scores indicating better physical performance (Guralnik et al., 1994).

The total SPPB score at 10 weeks (T1) will constitute the primary outcome of the trial.

#### 3.6.2 Secondary outcomes

##### 3.6.2.1 Handgrip strength

Handgrip strength will be assessed using a calibrated hand dynamometer following a standardized assessment protocol. Participants will perform three maximal isometric contractions with each hand, with standardized rest periods between attempts. The highest value obtained from the three trials for each hand will be recorded and expressed in kilograms (kg) (Vaishya et al., 2024). The highest value obtained with the dominant hand will be used as the primary handgrip strength measure for statistical analysis.

##### 3.6.2.2 Anxiety

Anxiety symptoms will be assessed using the Hamilton Anxiety Rating Scale (HAM-A), a 14-item clinician-rated instrument evaluating both psychological and somatic anxiety symptoms. Each item is scored from 0 to 4, yielding a total score ranging from 0 to 56, with higher scores indicating greater anxiety severity (Hamilton, 1959).

##### 3.6.2.3 Caregiver burden

Caregiver burden will be assessed using the Zarit Burden Interview (ZBI), a widely used instrument designed to evaluate the perceived impact of caregiving on emotional well-being, social functioning, and daily life. Higher scores indicate greater caregiver burden (Zarit et al., 1980; Martín et al., 1996).

#### 3.6.3 Intervention adherence, safety, and retention

Intervention adherence will be assessed by recording attendance at each scheduled session. For each participant, the number of sessions attended and the proportion of completed sessions relative to the total number of sessions prescribed for their allocated group will be calculated.

Safety and tolerability will be assessed through systematic monitoring and recording of adverse events throughout the intervention period. Before each training session, participants will be asked whether they have experienced any adverse events or new health problems since the previous session. The type, severity, and potential relationship of each reported adverse event to the intervention will be documented.

Participant retention will be assessed by recording the number and proportion of participants who complete the post-intervention and 3-month follow-up assessments. Reasons for withdrawal or loss to follow-up will be documented whenever available. If the person with dementia receiving care dies during the study, the participating caregiver will be withdrawn from the study, and the death will be recorded as the reason for withdrawal. All data collected up to the time of withdrawal will be retained for analysis in accordance with the intention-to-treat principle. Adequate adherence will be defined as attendance at ≥80% of the sessions prescribed for the participant’s allocated group.

### 3.7 Participant timeline

The study will be conducted over a 10-week intervention period, followed by a 3-month post-intervention follow-up. Assessments will be performed at three predefined time points: baseline (T0), immediately after completion of the 10-week intervention (T1), and at the 3-month follow-up (T2).

At baseline, participants will undergo eligibility screening, provide written informed consent, and complete the baseline assessment, including sociodemographic and caregiving-related characteristics, lower-extremity physical performance assessed using the Short Physical Performance Battery (SPPB), handgrip strength, anxiety symptoms assessed using the Hamilton Anxiety Rating Scale (HAM-A), and caregiver burden assessed using the Zarit Burden Interview (ZBI). Following completion of baseline assessments, participants will be randomly allocated to either the intervention or comparison group.

During the 10-week intervention period, participants allocated to the combined intervention group will receive one weekly functional strength training session and one weekly progressive muscle relaxation session, whereas participants allocated to the comparison group will receive one weekly progressive muscle relaxation session. Attendance, adherence, and adverse events will be recorded throughout the intervention period.

Immediately after completion of the intervention (T1), all outcome measures will be reassessed. The same assessments will be repeated at the 3-month follow-up (T2) to evaluate the maintenance of intervention effects over time. Participant retention and reasons for withdrawal or loss to follow-up will also be documented.

The schedule of enrolment, interventions, and assessments is summarized in Table 1 in accordance with the SPIRIT recommendations.

### 3.8 Sample size calculation

The sample size was calculated based on the primary outcome of the study, the total Short Physical Performance Battery (SPPB) score at 10 weeks. The calculation was based on detecting a minimum clinically meaningful between-group difference of 1 point in the total SPPB score, with an estimated standard deviation of 1.3 points, based on previous studies conducted in older and frail populations (Perera et al., 2006; Guralnik et al., 1994).

Assuming a two-sided significance level of α = 0.05 and a statistical power of 80%, a minimum of 27 participants per group will be required. To account for an anticipated dropout rate of approximately 13%, the target sample size will be increased to 31 participants per group, resulting in a total sample of 62 participants.

The sample size calculation was performed using G*Power software, version 3.1.9.7 (Heinrich Heine University Düsseldorf, Germany).

### 3.9 Data collection and management

All study data will be collected using standardized assessment procedures. Outcome assessments will be performed by the same independent researcher described above, who will not participate in any other study procedures that could reveal group allocation and will remain blinded to participants’ group assignment.

Each participant will be assigned a unique numerical identification code to ensure confidentiality. Personal identifying information will be stored separately from research data and will only be accessible to authorized members of the research team.

Study data will be entered into a secure electronic database specifically designed for the study. Data quality will be ensured through predefined validation rules, including range checks and consistency checks. Periodic data reviews will be conducted to identify missing, inconsistent, or implausible values. Any discrepancies identified during data entry or verification will be checked against the original source documents and corrected when appropriate.

Access to the final study dataset will be restricted to the principal investigators and authorized members of the research team. Data will be stored and retained in accordance with applicable institutional and data protection regulations.

Missing data will be documented, including the reasons for missingness whenever known. The handling of missing outcome data will follow the predefined statistical analysis plan.

### 3.10 Statistical analysis

Normality of quantitative variables will be assessed using the Shapiro–Wilk test, together with the visual inspection of histograms and Q–Q plots. Descriptive statistics will be reported as mean and standard deviation for approximately normally distributed variables, and as median and interquartile range for non-normally distributed variables. Categorical variables will be summarized using absolute and relative frequencies.

Baseline characteristics will be compared between groups using independent-samples Student’s *t*-tests or Mann–Whitney U tests for quantitative variables, as appropriate, and chi-square or Fisher’s exact tests for categorical variables. These baseline comparisons will be interpreted descriptively rather than used as a criterion for selecting covariates.

The primary analysis will follow the intention-to-treat principle and will include all randomized participants according to their assigned group.

The primary outcome will be analyzed using a linear mixed-effects model including post-intervention and 3-month follow-up SPPB scores as dependent observations. The model will include fixed effects for group, assessment time, the group-by-time interaction, and baseline SPPB score, together with a participant-specific random intercept.

Assessment time will be modelled as a categorical variable. The prespecified primary contrast will be the adjusted mean difference between groups in total SPPB score at 10 weeks. The adjusted between-group difference at the 3-month follow-up will be considered a secondary contrast.

Model assumptions will be evaluated by examining residual distributions, Q–Q plots, and residual-versus-fitted-value plots. If substantial violations remain after appropriate transformation or robust estimation procedures, an alternative pre-specified model appropriate for the outcome distribution will be used.

Continuous secondary outcomes, including handgrip strength, HAM-A score, and Zarit Burden Interview score, will be analysed using comparable mixed-effects models. Results will be reported as adjusted between-group mean differences with 95% confidence intervals and standardized effect sizes.

Secondary-outcome *p*-values will be adjusted for multiplicity using the Holm procedure. These analyses will be interpreted as supportive and exploratory relative to the primary SPPB analysis.

No formal hypothesis testing of within-group changes will be used to establish intervention effectiveness. Any within-group summaries will be presented descriptively.

Missing outcome data will be handled primarily through the mixed-effects model under a missing-at-random assumption. The amount, timing, and reasons for missing data will be reported by group. Multiple imputation will be conducted as a sensitivity analysis if the proportion or pattern of missing data warrants it. The imputation model will include treatment allocation, baseline outcome values, follow-up outcome values, and prespecified predictors of missingness.

A per-protocol analysis will be performed as a sensitivity analysis including participants who attend ≥80% of the sessions prescribed for their allocated group. All tests will be two-sided, with a significance level of 0.05 for the primary outcome. Analyses will be conducted using IBM SPSS Statistics software, version 30.

## 4 Discussion

The present protocol describes the design of a randomized controlled trial aimed at evaluating the effectiveness of a multicomponent intervention combining functional strength training and progressive muscle relaxation in informal caregivers of people living with dementia. The proposal is grounded in previous evidence highlighting the sustained negative impact of the caregiving role on functional capacity, physical health, and psychological well-being, as well as the need for comprehensive strategies that address these domains simultaneously (Cheng and Zhang, 2020; He et al., 2022).

Existing literature indicates that many interventions targeting informal caregivers have traditionally focused on isolated components, such as psychological support, education, or stress management, whereas others have prioritized physical exercise independently (Sun et al., 2022).

However, current evidence suggests that these partial approaches may be insufficient to address the complexity of caregiving, which involves intertwined physical, emotional, and functional demands. In this context, multicomponent interventions emerge as a promising alternative by enabling a more holistic approach to caregiver well-being (Huis In Het Veld et al., 2015; Kwok et al., 2023).

The program proposed in this study integrates a functional strength training component aimed at improving physical performance and muscle strength and reducing the risk of physical decline, together with a structured progressive muscle relaxation intervention designed to reduce anxiety, stress, and caregiver burden. This combined approach is based on the hypothesis that simultaneously targeting physical and psychological systems may produce broader and more sustainable benefits, promoting a healthier and more sustainable caregiving role over the medium and long term (Cardoso et al., 2025; Wang et al., 2024).

Furthermore, conducting the study in a real-world health and social care setting, such as the CREA in Salamanca, will allow the evaluation not only of the intervention’s effectiveness but also of its feasibility, safety, and acceptability. This aspect is particularly relevant, as one of the main limitations of existing evidence lies in the difficulty of translating findings from controlled studies into routine clinical and community practice (Perales-Puchalt et al., 2025; Pihet et al., 2024). The implementation of a structured, low-cost, and easily replicable program may facilitate the transfer of these findings to other care settings and community resources.

### 4.1 Strengths

Key strengths of this study include the use of validated and widely adopted instruments to assess lower-extremity physical performance, muscle strength, anxiety, and caregiver burden, as well as a rigorous methodological design that allows the additional effect of adding functional strength training to progressive muscle relaxation to be evaluated through comparison between the two intervention groups.

### 4.2 Limitations

Several limitations should be acknowledged. First, recruitment from a single specialized center may limit the generalizability of the findings and may result in selection of caregivers who are more engaged with support services. Second, participants and intervention providers cannot be blinded. Third, the combined intervention group will receive greater professional contact and overall intervention exposure than the comparison group. Which may contribute to the observed between-group effects. Fourth, participants with high baseline SPPB scores may demonstrate a ceiling effect. Fifth, the relatively short intervention and follow-up periods may limit conclusions regarding long-term maintenance. Sixth, group-based delivery may introduce clustering by intervention cohort or physiotherapist. Finally, changes in caregiving circumstances, outside physical activity, medication, or formal support during follow-up may influence the study outcomes.

### 4.3 Clinical implications

Overall, the findings derived from this randomized controlled trial may contribute to expanding the existing evidence on comprehensive interventions for informal caregivers of people with dementia, providing relevant information for the development of more effective, sustainable, and real-world-oriented support programs. This approach may have important implications not only for caregiver health and well-being but also for the quality of care delivered to people living with dementia.
