# Supplementary material for: Combined functional strength training and progressive muscle relaxation in informal caregivers of people with dementia: protocol for a randomized controlled trial
Source: Front Psychol. 2026 Sep 17;17:1944170. doi: 10.3389/fpsyg.2026.1944170 (PMC13627432; doi:10.3389/fpsyg.2026.1944170)
Supplement: Supplementary file 1 [file Data_Sheet_1.PDF]

ClinicalTrials.gov Protocol Registration and Results System (PRS) Receipt
Release Date: January 30, 2026

ClinicalTrials.gov ID: NCT07388186

Study Identification

Unique Protocol ID: STRENGTH_CAREGIVERS_ALZ

Brief Title: Benefits of Strength Training in Informal Caregivers of Patients With Alzheimer's Disease

Official Title: Effectiveness of a Combined Strength Training and Progressive Muscle Relaxation Program in Reducing Frailty in Informal Caregivers of People With Alzheimer's Disease: A Randomized Controlled Trial

Secondary IDs:

Study Status

Record Verification: January 2026

Overall Status: Not yet recruiting

Study Start: March 2026 [Anticipated]

Primary Completion: July 2026 [Anticipated]

Study Completion: September 2026 [Anticipated]

Sponsor/Collaborators

Sponsor: University of Salamanca

Responsible Party: Principal Investigator
Investigator: Andrea Calleja Caballero [ACallejaCaballero]
Official Title: Physical therapist
Affiliation: University of Salamanca

Collaborators: Colegio Profesional de Fisioterapeutas de Castilla y León
CRE Alzheimer del Imserso

Oversight

U.S. FDA-regulated Drug: No

U.S. FDA-regulated Device: No

U.S. FDA IND/IDE: No

Human Subjects Review: Board Status: Approved
Approval Number: 1468
Board Name: Bioethics Committee of the University of Salamanca
Board Affiliation: University of Salamanca
Phone:
Email: ceinves@usal.es

Address:

Data Monitoring: No

Study Description

Brief Summary: Informal caregivers of people with Alzheimer's disease experience high physical and psychological burden, increasing their risk of frailty, anxiety, and caregiver burnout. This randomized controlled trial aims to evaluate the effectiveness of a combined strength training and progressive muscle relaxation program compared with progressive muscle relaxation alone. The intervention will last 10 weeks, with assessments conducted at baseline, post-intervention, and at a 3-month follow-up. Primary outcomes include frailty, anxiety, and caregiver burden.

Detailed Description: Alzheimer's disease is associated with long-term caregiving demands that expose informal caregivers to sustained physical and psychological stress. This chronic exposure may contribute to functional decline, increased vulnerability to frailty, and reduced overall well-being, highlighting the need for accessible, non-pharmacological interventions targeting caregiver health.

The present study adopts a parallel-group randomized controlled design in which participants are allocated to one of two intervention arms. One group will participate in structured progressive muscle relaxation sessions, while the other will receive a combined intervention integrating functional strength training with progressive muscle relaxation. The intervention protocol has been designed to be feasible, low-cost, and easily implementable in community and clinical settings.

Assessments will be conducted at multiple time points to examine changes in physical performance and psychological status over time. Standardized performance-based measures and validated self-report instruments will be used to capture functional and psychosocial outcomes, allowing for a comprehensive evaluation of the intervention effects across short- and medium-term follow-up periods.

Conditions

Conditions: Frailty
Caregiver Burden
Anxiety

Keywords: Frailty
Strength
Caregiver
Anxiety
Burden
Alzheimer disease

Study Design

Study Type: Interventional

Primary Purpose: Treatment

Study Phase: N/A

Interventional Study Model: Parallel Assignment

Participants will be randomly assigned to one of two parallel intervention arms and will receive either progressive muscle relaxation alone or a combined program of functional strength training and progressive muscle relaxation over a 10-week period.

Number of Arms: 2

Masking: Single (Outcomes Assessor)

Allocation: Randomized

Enrollment: 62 [Anticipated]

Arms and Interventions

Arms	Assigned Interventions
Active Comparator: Progressive Muscle Relaxation<br>Participants in this arm will receive one weekly session of progressive muscle relaxation over a 10-week period. Each session will last approximately 45 minutes and will include diaphragmatic breathing, Jacobson’s progressive muscle relaxation technique, controlled breathing exercises, and a brief cooldown period. Sessions will be supervised by a trained physiotherapist.	Behavioral: Progressive Muscle Relaxation<br>Progressive muscle relaxation will be delivered in weekly supervised sessions over a 10-week period. Each session will last approximately 45 minutes and will include diaphragmatic breathing, systematic muscle contraction and relaxation following Jacobson's technique, controlled breathing exercises, and a brief cooldown period. The intervention will be administered by a trained physiotherapist in a group setting.
Experimental: Strength Training plus Progressive Muscle Relaxation<br>Participants in this arm will receive a combined intervention consisting of one weekly session of progressive muscle relaxation and one weekly session of functional strength training over a 10-week period. Strength training sessions will focus on upper limb, lower limb, and core muscle groups using progressive resistance. Each session will last approximately 45 minutes and will be supervised by a trained physiotherapist.	Behavioral: Progressive Muscle Relaxation<br>Progressive muscle relaxation will be delivered in weekly supervised sessions over a 10-week period. Each session will last approximately 45 minutes and will include diaphragmatic breathing, systematic muscle contraction and relaxation following Jacobson's technique, controlled breathing exercises, and a brief cooldown period. The intervention will be administered by a trained physiotherapist in a group setting.<br>Behavioral: Functional Strength Training<br>Functional strength training will be delivered in weekly supervised sessions over a 10-week period. Sessions will last approximately 45 minutes and will focus on improving upper limb, lower limb, and core muscle strength using progressive resistance exercises. Training intensity will be gradually increased according to participant tolerance and performance. All sessions will be supervised by a trained physiotherapist.

Outcome Measures

Primary Outcome Measure:

1. Frailty

   Frailty will be assessed using the Short Physical Performance Battery (SPPB) and handgrip strength measured with a hand dynamometer.

   The SPPB includes balance, gait speed, and lower-limb strength components. The score is obtained by summing the results of three performance-based tests: balance, gait speed over 4 meters, and the five-times sit-to-stand chair test. The total score ranges from 0 to 12 points, where 0 represents the worst performance and 12 the best performance. Lower scores (generally <10) indicate frailty and increased risk of falls. Performance is classified as follows: 0–3 (disabled), 4–6 (frail), 7–9 (prefrail), and 10–12 (independent).

Handgrip strength will be measured using a hand dynamometer, and the absolute grip strength value will be recorded. The Fried frailty phenotype includes handgrip strength (dynamometry) as one of the five key criteria for identifying frailty in older adults. Cut-off values indicative of muscle weakness, adjusted for sex and body mass

[Time Frame: Baseline, 3-month follow-up and immediately after completion of the intervention (at 10 weeks).]

Secondary Outcome Measure:

2. Anxiety
   Anxiety symptoms will be assessed using the Hamilton Anxiety Rating Scale (HAM-A), a validated clinician-rated questionnaire evaluating psychological and somatic anxiety symptoms. HAM-A is scored on a scale of 0 (not present) to 4 (severe), with a total score range of 0–56, where <17 indicates mild severity, 18–24 mild to moderate severity and 25–30 moderate to severe.

   [Time Frame: Baseline, 3-month follow-up and immediately after completion of the intervention (at 10 weeks)]
3. Caregiver burden
   Caregiver burden will be assessed using the Zarit Burden Interview (ZBI), a validated self-report questionnaire measuring perceived burden in informal caregivers. The total score is calculated as the sum of all items, with a possible range from 22 to 110. Each item is rated using a 5-point Likert-type scale, with response options ranging from “never” to “almost always”, scored from 1 to 5.

   [Time Frame: Baseline, 3-month follow-up and immediately after completion of the intervention (at 10 weeks)]

Other Pre-specified Outcome Measures:

4. Adherence to the intervention
   Adherence will be assessed by recording the number of sessions attended and the percentage of completed sessions for each participant.

   [Time Frame: Throughout the 10-week intervention period]
5. Adverse events
   Adverse events or incidents occurring during the intervention sessions, such as musculoskeletal discomfort, dizziness, or other exercise-related events, will be recorded and monitored.

   [Time Frame: Throughout the 10-week intervention period]

Eligibility

Minimum Age: 18 Years

Maximum Age:

Sex: All

Gender Based: No

Accepts Healthy Volunteers: Yes

Criteria: Inclusion Criteria:

- Adults aged 18 years or older
- Informal caregivers of a person with Alzheimer’s disease
- Providing regular care for at least 10 hours per week
- Ability to attend intervention sessions
- Ability to understand and follow instructions
- Provision of written informed consent

Exclusion Criteria:

- Acute illness or medical conditions contraindicating participation in strength exercise
- Participation in structured exercise programs that may interfere with study outcomes
- Severe or uncontrolled psychiatric disorders

• Physical limitations preventing completion of functional assessments

Contacts/Locations

Central Contact Person: Andrea Calleja Caballero, Principal Investigator
Telephone: +34 616957689
Email: andreacallejac@usal.es

Central Contact Backup:

Study Officials:

Locations: **Spain**
Universidad de Salamanca
Salamanca, Spain, 37007
Contact: Fátima Perez-Robledo +34 619 885 479 fatima_pr@usal.es

IPDSharing

Plan to Share IPD: No
De-identified individual participant data will not be shared outside the study team due to ethical, privacy, and data protection considerations.

References

Citations:

Links:

Available IPD/Information:
