# Supplementary material for: Combined functional strength training and progressive muscle relaxation in informal caregivers of people with dementia: protocol for a randomized controlled trial
Source: Front Psychol. 2026 Sep 17;17:1944170. doi: 10.3389/fpsyg.2026.1944170 (PMC13627432; doi:10.3389/fpsyg.2026.1944170)
Supplement: Supplementary file 2 [file Data_Sheet_2.PDF]

Functional strength training program for informal caregivers of people with Alzheimer’s disease.

**Why:**	The strength training component was designed to improve physical performance, enhance muscle strength, and prevent or reduce physical frailty in informal caregivers exposed to sustained physical demands.
**What (material):**	Materials included elastic resistance bands of varying resistance levels, adjustable dumbbells (1–5 kg), stability balls, and standardized seating equipment. Sessions were delivered using equipment provided and maintained by the research team in adapted exercise facilities.
**What (procedures):**	The strength training sessions were structured into three phases: warm-up (joint mobility and low-intensity activation exercises), main phase (functional multi-joint exercises replicating activities of daily living such as sit-to-stand, step-ups, pushing, pulling, and balance tasks), and cool-down. Exercises were performed in sets of 8–12 repetitions (1–3 sets) with progressive adjustment of intensity.
**Who provided:**	The program was delivered by licensed physiotherapists with at least two years of clinical experience in therapeutic exercise.
**How (mode of delivery; individual or group):**	The intervention was delivered face-to-face in small group formats under direct supervision of a physiotherapist.
**Where:**	The intervention was conducted at the Centro de Referencia Estatal de Atención a Personas con Enfermedad de Alzheimer y otras Demencias (CREA) in Salamanca, Spain, in a controlled and adapted exercise environment.
**When and how much:**	The strength training program lasted 10 weeks and was delivered once weekly to participants allocated to the experimental group. Each session lasted approximately 45–60 minutes.
**Tailoring:**	Exercises were individually adapted according to each participant’s functional level. Intensity and progression were guided by perceived exertion, functional performance, and predefined safety criteria. Progression decisions were based on participant tolerance, correct technical execution, and absence of adverse symptoms.
**How well (planned):**	Adherence will be monitored by recording attendance at each scheduled session. Sessions will be delivered according to a predefined standardized protocol under direct supervision of a physiotherapist to ensure intervention fidelity. Participants will receive reminders and motivational support when necessary to enhance adherence.
