# Supplementary material for: Combined functional strength training and progressive muscle relaxation in informal caregivers of people with dementia: protocol for a randomized controlled trial
Source: Front Psychol. 2026 Sep 17;17:1944170. doi: 10.3389/fpsyg.2026.1944170 (PMC13627432; doi:10.3389/fpsyg.2026.1944170)
Supplement: Supplementary file 3 [file Data_Sheet_3.PDF]

TIDieR

Combined functional strength training and progressive muscle relaxation program for informal caregivers of people with Alzheimer’s disease.

Why: The intervention integrates functional strength training and progressive muscle relaxation to address physical frailty and psychological burden in informal caregivers of people with Alzheimer’s disease.

What (material): Materials included elastic resistance bands of varying resistance levels, adjustable dumbbells (1–5 kg), stability balls, standardized seating equipment, and a calibrated digital hand dynamometer. Progressive muscle relaxation sessions were delivered using a structured written protocol with standardized verbal instructions provided by the physiotherapist.

What (procedures): The strength training sessions were structured into three phases: warm-up (joint mobility and low-intensity activation exercises), main phase (functional multi-joint exercises replicating activities of daily living such as sit-to-stand, step-ups, pushing, pulling, and balance tasks), and cool-down. Exercises were performed in sets of 8–12 repetitions (1–3 sets) with progressive adjustment of intensity.
The progressive muscle relaxation sessions followed a structured protocol based on sequential contraction and relaxation of major muscle groups combined with controlled breathing and guided verbal instructions delivered by the physiotherapist.

Who provided: The intervention was delivered by licensed physiotherapists with at least two years of clinical experience in therapeutic exercise and prior training in progressive muscle relaxation techniques.

How (mode of delivery; individual or group): The intervention was delivered face-to-face in small group formats under direct supervision of a physiotherapist.

Where: The intervention was conducted at the Centro de Referencia Estatal de Atención a Personas con Enfermedad de Alzheimer y otras Demencias (CREA) in Salamanca, Spain, in a controlled and adapted exercise environment.

When and how much: The intervention lasted 10 weeks. The experimental group attended two weekly sessions (one session of functional strength training and one session of progressive muscle relaxation), whereas the control group attended one weekly session of progressive muscle relaxation. Strength training sessions lasted approximately 45–60 minutes, and progressive muscle relaxation sessions lasted approximately 30–45 minutes.

Tailoring: Exercises were individually adapted according to each participant’s functional level. Intensity and progression were guided by perceived exertion, functional performance, and predefined safety criteria. Progression decisions were based on participant tolerance, correct technical execution, and absence of adverse symptoms.

How well (planned): Adherence will be monitored by recording attendance at each scheduled session. Sessions will be delivered according to a predefined standardized protocol under direct supervision of a physiotherapist to ensure intervention fidelity. Participants will receive reminders and motivational support when necessary to enhance adherence.

Combined functional strength training and progressive muscle relaxation program for informal caregivers of people with Alzheimer’s disease.
