# Supplementary material for: Combined functional strength training and progressive muscle relaxation in informal caregivers of people with dementia: protocol for a randomized controlled trial
Source: Front Psychol. 2026 Sep 17;17:1944170. doi: 10.3389/fpsyg.2026.1944170 (PMC13627432; doi:10.3389/fpsyg.2026.1944170)
Supplement: Supplementary file 4 [file Data_Sheet_4.PDF]

TIDieR

Progressive muscle relaxation program for informal caregivers of people with Alzheimer’s disease.

**Por qué:**	The progressive muscle relaxation component was designed to reduce anxiety, stress, and caregiver burden by promoting physiological relaxation, body awareness, and emotional self-regulation.
**Qué (material):**	The intervention was delivered using a structured written protocol based on Jacobson’s progressive muscle relaxation technique. Standardized verbal instructions were provided by the physiotherapist in a calm and controlled environment.
**Qué (procedimientos):**	Sessions followed a structured sequence of systematic contraction and relaxation of major muscle groups, progressing from distal to proximal regions. The procedure incorporated controlled breathing exercises and guided verbal instructions delivered by the physiotherapist to facilitate relaxation and stress reduction.
**Quién proporcionó:**	The program was delivered by licensed physiotherapists with prior training in progressive muscle relaxation techniques.
**Cómo (modo de prestación; individual o grupal):**	The intervention was delivered face-to-face in small group formats under direct supervision of a physiotherapist.
**Dónde:**	The intervention was conducted at the Centro de Referencia Estatal de Atención a Personas con Enfermedad de Alzheimer y otras Demencias (CREA) in Salamanca, Spain, in a controlled and adapted exercise environment.
**Cuándo y cuánto:**	The program lasted 10 weeks. Participants in the experimental group attended one weekly relaxation session in combination with strength training, while participants in the control group attended one weekly relaxation session as a standalone intervention. Each session lasted approximately 30–45 minutes.
**Adaptación:**	The pace and verbal guidance of the relaxation exercises were adapted according to participants’ comfort, tolerance, and individual needs to ensure safety and effectiveness.
**Cuán bien (planificada):**	Adherence will be monitored through attendance records. Sessions will follow a predefined structured relaxation protocol with standardized verbal guidance delivered by the physiotherapist to ensure intervention fidelity.
